# A late awakening: delayed functional transformation of a silent corticotroph adenoma into Cushing disease

**DOI:** 10.1210/jcemcr/luag301

**Published:** 2026-10-01

**Authors:** Nina Maria Fanaropoulou, Shehzad Basaria, David M Meredith, Whitney W Woodmansee, Timothy R Smith, George A Stamatiades

**Affiliations:** Division of Endocrinology, Diabetes and Metabolism, Department of Medicine, Mass General Brigham, Harvard Medical School, Boston, MA 02115, USA; Department of Internal Medicine, Miller School of Medicine, University of Miami; Jackson Memorial Hospital, Miami, FL 33136, USA; Division of Endocrinology, Diabetes and Metabolism, Department of Medicine, Mass General Brigham, Harvard Medical School, Boston, MA 02115, USA; Department of Pathology, Mass General Brigham, Harvard Medical School, Boston, MA 02115, USA; Division of Endocrinology, Diabetes and Metabolism, Department of Medicine, University of Florida, Gainesville, FL 32611, USA; Pituitary Center, Department of Neurosurgery, Mass General Brigham, Harvard Medical School, Boston, MA 02115, USA; Division of Endocrinology, Diabetes and Metabolism, Department of Medicine, Mass General Brigham, Harvard Medical School, Boston, MA 02115, USA

**Keywords:** Cushing disease, corticotroph adenoma, pituitary, transcription factors

## Abstract

Silent corticotroph adenomas (SCAs) are a biologically distinct subset of nonfunctioning pituitary adenomas (NFPAs) characterized by corticotroph lineage without clinical or biochemical evidence of hypercortisolism. Although SCAs exhibit more aggressive growth behavior than other NFPAs, delayed transformation into overt adrenocorticotropic hormone (ACTH)-dependent hypercortisolism is rare. We describe a woman in her early 30s who presented with headaches and visual field deficits and was found to have a pituitary macroadenoma. Initial endocrine evaluation revealed mild hyperprolactinemia attributed to stalk effect, without evidence of hormonal hypersecretion. She underwent transsphenoidal surgery, and pathology was interpreted as an NFPA based on negative immunostaining. Transcription factor analysis was not performed. Postoperative imaging demonstrated a stable 5-mm residual lesion. More than 2 decades after surgery, progressive ACTH elevation emerged despite continued radiographic stability and preceded the development of clinical hypercortisolism by several years. Biochemical testing confirmed ACTH-dependent Cushing disease. Repeat surgery was performed and surgical pathology showed a T-box transcription factor-positive corticotroph adenoma with sparsely granulated ACTH expression. This case highlights the potential for very late functional transformation of SCAs and underscores the importance of long-term clinical and biochemical surveillance even in asymptomatic patients with stable residual disease on imaging.

## Introduction

Silent corticotroph adenomas (SCAs) are a distinct subset of nonfunctioning pituitary adenomas (NFPAs) characterized by corticotroph differentiation in the absence of clinical or biochemical evidence of hypercortisolism [1]. Historically, these tumors were identified through adrenocorticotropic hormone (ACTH) immunopositivity; however, advances in transcription factor profiling, particularly T-box transcription factor (TPIT) expression, have improved recognition of corticotroph-lineage tumors that were previously classified as nonfunctioning or null-cell adenomas [1].

SCAs account for ∼20% of NFPAs and exhibit distinct clinical and biological features, including a slight female predominance [2]. They are more likely to present with visual defects, exhibit invasive growth patterns, suprasellar extension, cavernous and sphenoid sinuses involvement and hypopituitarism. Notably, SCAs are associated with earlier and more frequent recurrence, often within 4 years [2], driven by both intrinsic tumor biology and challenges in achieving complete resection due to vascular and parasellar involvement [3, 4]. As a result, SCAs are increasingly recognized as a higher-risk pituitary adenoma subtype with a clinical course that may differ substantially from other NFPAs.

In addition to structural aggressiveness, SCAs possess the potential for functional transformation to overt ACTH-dependent Cushing disease. Such transformation has been reported in ∼4% to 9% of retrospective cohorts [1, 5], although these estimates may be conservative because earlier studies predated routine transcription factor profiling. Proposed mechanisms behind this transformation include altered proopiomelanocortin (POMC) processing, increased prohormone convertase 1/3 activity, epigenetic changes affecting ACTH regulation, and progressive acquisition of secretory capacity within residual corticotroph tumor cells [5]. Transformation after prolonged radiographic stability is particularly rare, and the long-term endocrine potential of SCAs remains poorly defined. Consequently, current surveillance strategies are focused primarily on radiographic monitoring, while the role of long-term biochemical surveillance remains uncertain.

In this report, we describe a case of a pituitary adenoma, initially classified as nonfunctioning, that subsequently underwent clinical and biochemical transformation into ACTH-dependent Cushing disease more than 2 decades after initial treatment, despite sustained radiographic stability. This case underscores the potential for delayed functional evolution in SCAs, highlights the importance of modern tumor classification, and raises important considerations regarding long-term surveillance strategies even in patients with structurally stable residual disease.

## Case presentation

A 31-year-old woman presented with headaches and progressive visual disturbances. Formal visual field testing demonstrated bitemporal hemianopsia, and magnetic resonance imaging (MRI) revealed a 12 × 13 × 14 mm sellar mass with suprasellar extension with compression of the optic chiasm, consistent with a pituitary macroadenoma (Fig. 1).

**Figure 1:**
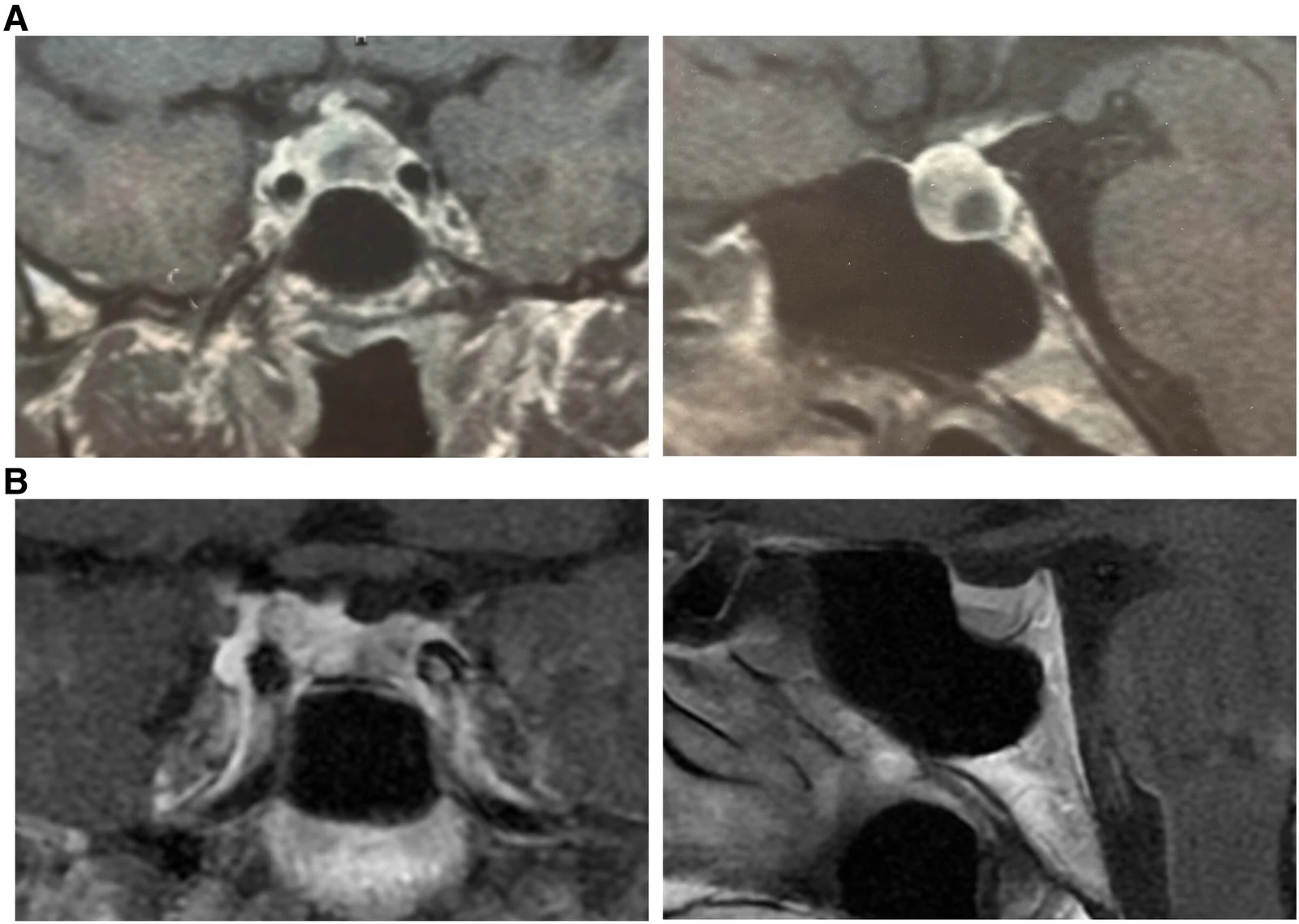
Radiographic evolution of the pituitary adenoma. (A) Coronal and sagittal pituitary magnetic resonance images at initial presentation demonstrating a macroadenoma with suprasellar extension and compression of the optic chiasm. (B) Corresponding coronal and sagittal images obtained more than 2 decades after initial transsphenoidal surgery demonstrating stable residual lesion without significant interval growth.

## Diagnostic assessment

Initial endocrine evaluation revealed mild hyperprolactinemia 32 ng/mL (SI: 32 μg/L) (reference range, 4.8-23.3 ng/mL [SI: 4.8-23.3 μg/L]) attributed to the stalk effect, without clinical features suggestive of overt hypercortisolism, evidence of other pituitary hormone hypersecretion or hypopituitarism. An overnight 1-mg dexamethasone suppression test was not performed at initial presentation. Dopamine agonist therapy did not reduce tumor size. The patient subsequently underwent transsphenoidal surgery.

Histopathologic examination of the resected tumor demonstrated a pituitary adenoma that was negative for pituitary hormone immunostaining. As transcription factor analysis was not available at that time, the lesion was classified as an NFPA. Postoperatively, MRI demonstrated a residual hypoenhancing lesion measuring ∼5 mm, consistent with subtotal resection. Serial MRI over the subsequent 2 decades did not demonstrate any interval growth or change in radiographic appearance.

More than 2 decades after the initial surgery, routine biochemical surveillance demonstrated progressively increasing ACTH levels, despite no change in clinical status and continued radiographic stability of the residual lesion (Fig. 2). Serum ACTH levels gradually increased from 38 pg/mL (SI: 8.4 pmol/L) (reference range, 7-63 pg/mL [SI: 1.5-13.9 pmol/L]) to a peak of 103 pg/mL (SI: 22.7 pmol/L) (Table 1) with morning serum cortisol levels also progressively increasing from 17.1 μg/dL (SI: 472 nmol/L) (reference range, 5-25 μg/dL [SI: 138-690 nmol/L]) to 24.7 μg/dL (SI: 682 nmol/L). Two years after these biochemical changes manifested, the patient's clinical status also changed as she developed progressive weight gain of 20 pounds and reported increased irritability, raising concern for evolving clinical hypercortisolism.

**Figure 2:**
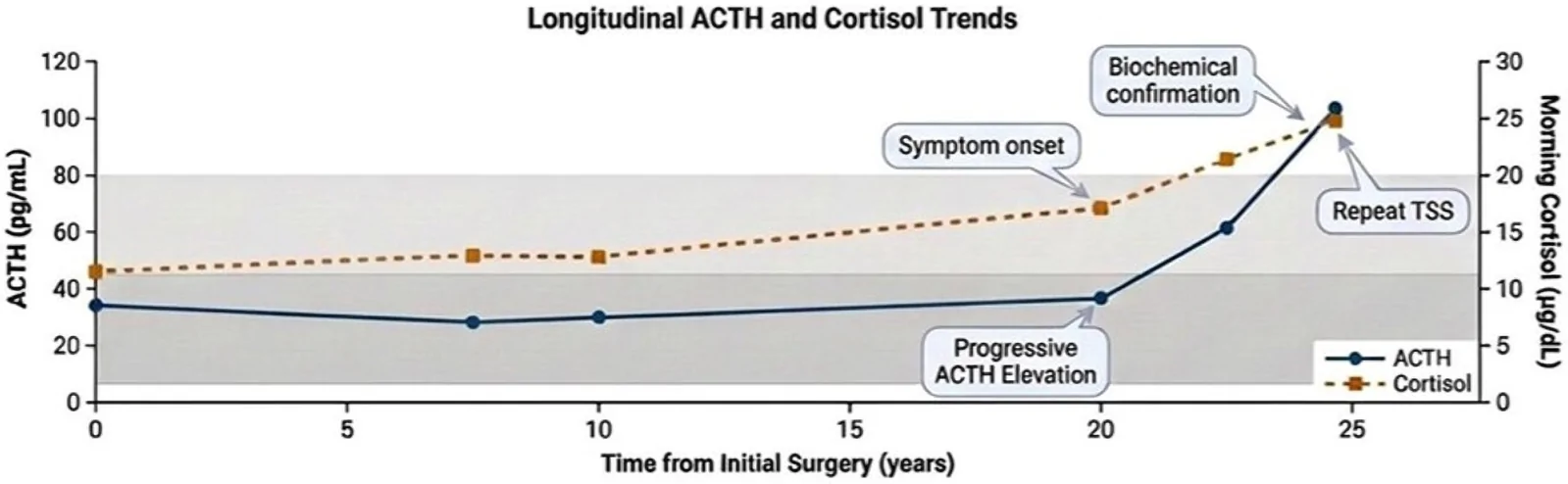
Longitudinal biochemical and clinical evolution. Progressive elevation in plasma adrenocorticotropic hormone (ACTH) concentrations and morning serum cortisol levels over time following initial transsphenoidal surgery, despite prolonged radiographic stability of the residual sellar lesion. Progressive ACTH elevation preceded development of clinical symptoms and subsequent biochemical confirmation of ACTH-dependent hypercortisolism.

**Table 1:** Dynamic hypercortisolism testing results

Test	Result	Reference range
24 hours UFC	60 μg/24 hours (166 nmol/24 hours)	<50 μg/24 hours (<138 nmol/24 hours)
Late-night salivary cortisol	143 ng/dL (3.95 nmol/L)	<100 ng/dL (<2.76 nmol/L)
1-mg overnight DST ACTH	40 pg/mL (8.8 pmol/L)	Not applicable
1-mg overnight DST cortisol	6.4 μg/dL (177 nmol/L)	<1.8 μg/dL (<50 nmol/L)

Values in parenthesis are International System of Units (SI).

Abbreviations: ACTH, adrenocorticotropic hormone; DST, dexamethasone suppression test; UFC, urinary free cortisol.

Further endocrine evaluation demonstrated elevated 24-hour urinary free cortisol of 60 μg/24 hours (SI: 166 nmol/24 hours) (reference range, <50 μg/24 hours [SI: <138 nmol/24 hours]) and elevated late-night salivary cortisol of 143 ng/dL (SI: 3.95 nmol/L) (reference range, < 100 ng/dL [SI: <2.8 nmol/L]). An overnight 1-mg dexamethasone suppression test demonstrated persistent elevation of both ACTH (40 pg/mL [SI: 8.8 pmol/L]) and serum cortisol (6.4 μg/dL [SI: 177 nmol/L]) (Table 1). These findings were consistent with ACTH-dependent hypercortisolism.

## Treatment

Following confirmation of ACTH-dependent Cushing disease, the patient underwent repeat transsphenoidal surgery for management of Cushing disease. Histopathologic evaluation of the resected tissue demonstrated a TPIT-positive corticotroph adenoma with sparsely granulated ACTH expression (Fig. 3). Reticulin staining showed preserved acinar architecture, and a MIB-1 labeling index was low at ∼1%. No significant mitotic activity or high-risk histopathologic features were identified.

**Figure 3:**
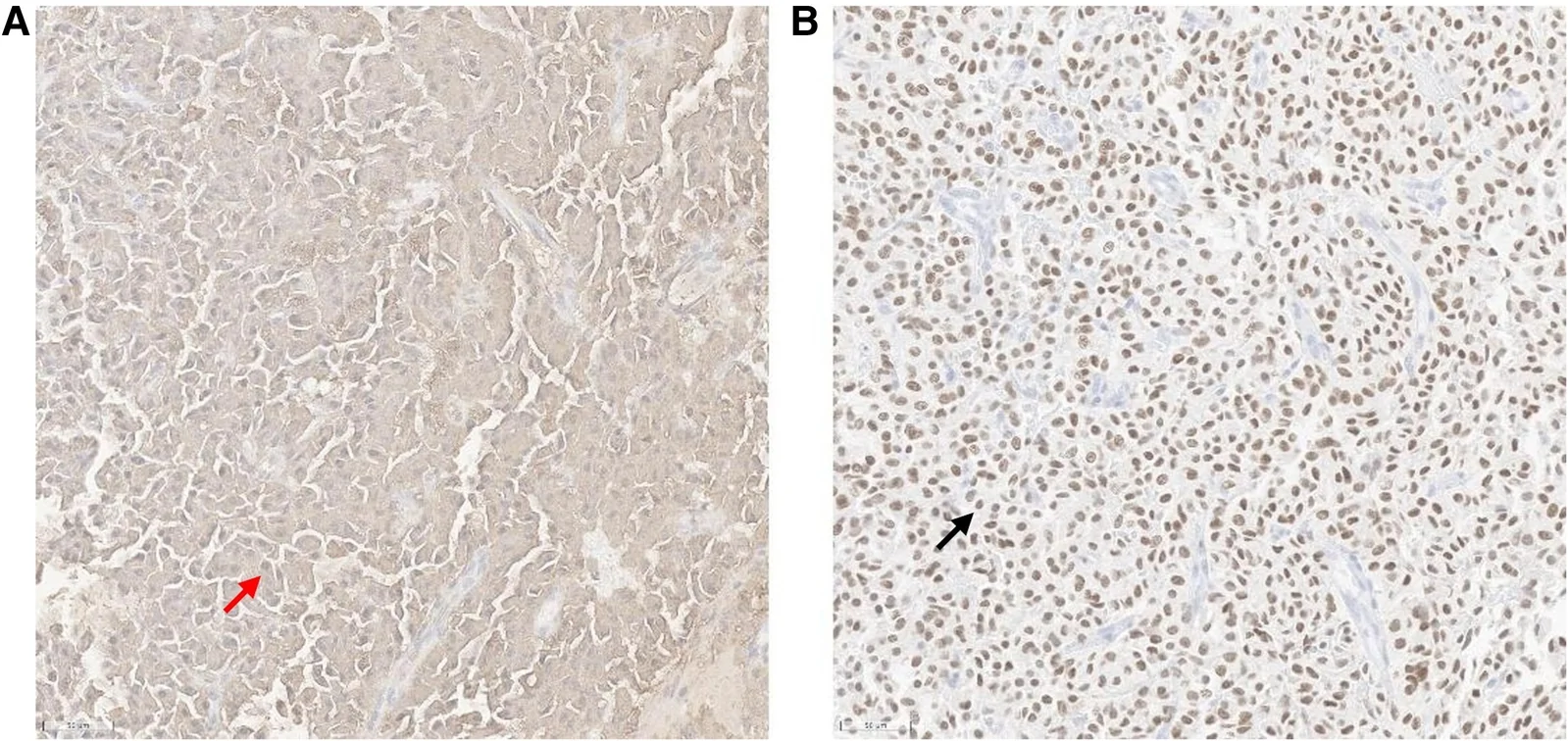
Histopathologic evaluation of the corticotroph adenoma at repeat surgery. (A) Weak and patchy cytoplasmic ACTH immunoreactivity consistent with sparsely granulated corticotroph differentiation (arrow) and (B) Diffuse nuclear TPIT immunoreactivity confirming corticotroph lineage within the adenoma cells (arrow). Original magnification ×200; scale bar, 50 μm.

## Outcome and follow-up

Postoperatively, the patient developed secondary adrenal insufficiency requiring hydrocortisone replacement therapy. At 5-month follow-up, the patient remained on physiologic hydrocortisone replacement, experienced weight loss, and reported improvement in neuropsychiatric symptoms. Follow-up MRI demonstrated expected postoperative changes without evidence of residual or recurrent disease.

## Discussion

This case describes functional transformation of a presumed SCA into overt Cushing disease more than 2 decades after initial treatment despite sustained radiographic stability. To our knowledge, this represents one of the longest reported intervals between initial diagnosis and overt hypercortisolism in a presumed SCA.

At initial diagnosis, the tumor was classified as an NFPA based on negative pituitary hormone immunostaining, as transcription factor analysis was unavailable [6]. However, the transition from absent ACTH immunoreactivity in the original specimen to weak ACTH expression in the current specimen is consistent with the functional transformation observed clinically and biochemically. With contemporary pathologic classification, including routine use of TPIT immunostaining as recommended in the World Health Organization classification of pituitary tumors [7], this lesion would likely have been identified as a silent corticotroph adenoma. This distinction is clinically meaningful, as SCAs are now recognized as a biologically distinct subgroup of pituitary adenomas with clinical behavior that differs substantially from other nonfunctioning tumors. Definitive confirmation that the original tumor represented an SCA is limited by the absence of transcription factor analysis from the initial surgical specimen. Importantly, reliance on ACTH immunostaining alone may miss a substantial proportion of corticotroph-lineage tumors. In a series of 105 TPIT-positive SCAs, 62.9% were ACTH-immunonegative, highlighting the diagnostic value of transcription factor profiling [8]. Compared with ACTH-positive SCAs, ACTH-negative tumors demonstrated lower circulating ACTH concentrations, a higher prevalence of multiple microcysts on MRI, and lower Ki-67 expression, whereas no significant differences were observed in the rates of suprasellar extension, cavernous sinus invasion, or gross-total resection [8]. These findings underscore the complementary role of TPIT immunostaining in establishing corticotroph lineage when ACTH immunoreactivity is absent.

Although SCAs are recognized as a higher-risk subgroup of NFPAs with increased invasive potential and postoperative recurrence [9], delayed endocrine transformation has received comparatively less attention. Functional transformation of SCAs into clinically functioning ACTH-secreting tumors causing Cushing disease has been described but appears to be uncommon. Retrospective cohorts have reported transformation rates ranging from 6.8% to 9% [5, 10, 11], although these estimates are likely conservative given historical limitations in tumor classification prior to widespread incorporation of transcription factor profiling. Reported cases typically occur within several years of diagnosis and are frequently associated with tumor progression or radiographic recurrence, with median time to transformation reported at 30 months (range 6 months to 7 years) [10, 11]. To our knowledge, delayed cases extending beyond a decade have not been reported previously.

In contrast, the present case demonstrated more than 2 decades of stable residual disease before biochemical evidence of hypercortisolism emerged. Progressive ACTH elevation preceded overt clinical disease by several years, providing a unique longitudinal view of endocrine evolution from clinically silent corticotroph disease to overt ACTH-dependent hypercortisolism. The absence of radiographic progression throughout this period argues against conventional tumor recurrence as the primary mechanism underlying the patient's clinical evolution. Furthermore, repeat pathology demonstrated preserved acinar architecture, low proliferative index, and absence of high-risk histopathologic features, findings that further support delayed phenotypic conversion rather than aggressive tumor regrowth.

The mechanisms underlying functional transformation remain incompletely understood. Reduced expression of prohormone convertase 1/3 (PC1/3), a key enzyme involved in cleavage of POMC into biologically active ACTH, has been implicated in maintenance of the silent corticotroph phenotype. In one study, low PC1/3 expression was identified in 3 SCAs that subsequently underwent functional transformation, with marked upregulation observed following progression to overt Cushing disease [5]. Interestingly, low expression was also observed in 14 nontransforming SCAs, suggesting that reduced PC1/3 may represent a characteristic feature of the silenced state. Increasing PC1/3 activity may enhance cleavage of POMC into biologically active ACTH, thereby driving transition to overt hypercortisolism. Sequential hypomethylation of a second POMC promoter, typically methylated in SCAs, has recently been associated with functional transformation and may represent an additional mechanism driving ACTH hypersecretion [12]. Collectively, these findings suggest that transformation may reflect gradual molecular and epigenetic reprogramming within residual corticotroph-lineage tumor cells rather than an abrupt biologic change.

An additional notable feature of this case is the prolonged biochemical evolution preceding overt clinical disease. Progressive ACTH elevation preceded clinically apparent hypercortisolism by several years, providing a unique longitudinal view of the gradual endocrine evolution of this tumor. This observation suggests that functional transformation may remain clinically occult for an extended period and may be detectable biochemically before overt manifestations of hypercortisolism develop. Because surveillance of NFPAs remains heavily weighted toward radiographic monitoring, this case demonstrates that prolonged structural stability does not necessarily indicate biologic quiescence. Periodic biochemical assessment may therefore have value in patients with known or suspected SCAs, particularly those with residual disease, to identify evolving corticotroph activity despite stable imaging.

This report has several limitations. Definitive confirmation that the original lesion represented an SCA is limited by the absence of transcription factor analysis from the initial surgical specimen, and retrospective immunohistochemical reassessment of the original tumor was not possible. In addition, an overnight 1-mg dexamethasone suppression test was not performed at the initial presentation; therefore, the presence of subtle biochemical hypercortisolism at that time cannot be definitively excluded. Nevertheless, the absence of clinical features of hypercortisolism at initial presentation, followed by prolonged radiographic stability, delayed biochemical progression, and subsequent demonstration of a TPIT-positive corticotroph adenoma strongly supports the case of delayed functional transformation.

In summary, this case expands the current understanding of SCA biology by demonstrating that prolonged structural stability does not preclude delayed functional transformation. The exceptionally long latency between initial diagnosis and overt Cushing disease suggests that residual corticotroph-lineage tumors may retain latent secretory potential for decades. These findings reinforce the importance of contemporary pathologic classification and support incorporation of long-term clinical and biochemical surveillance into follow-up strategies for patients with SCAs, even in the setting of stable residual disease on imaging.

## Learning points

- SCAs may undergo functional transformation to ACTH-dependent Cushing disease despite prolonged radiographic stability
- Stable imaging does not exclude endocrine progression, highlighting the value of long-term biochemical surveillance.
- Modern transcription factor–based pathology, including TPIT immunostaining, improves recognition of corticotroph-lineage tumors.

## Data Availability

The data underlying this article are included within the article. Additional deidentified data are available from the corresponding author upon reasonable request.
